# The Reduction in Microtubule Arrays Caused by the Dysplasia of the Non-Centrosomal Microtubule-Organizing Center Leads to a Malformed Organ of Corti in the Cx26-Null Mouse

**DOI:** 10.3390/biomedicines10061364

**Published:** 2022-06-09

**Authors:** Yue Qiu, Kai Xu, Le Xie, Sen Chen, Yu Sun

**Affiliations:** 1 Institute of Otorhinolaryngology, Tongji Medical College, Huazhong University of Science and Technology, Wuhan 430022, China; 2020xh0034@hust.edu.cn (Y.Q.); m201675568@hust.edu.cn (K.X.); 2019xh0094@hust.edu.cn (L.X.); 2 Department of Otolaryngology, Nanchang University, Nanchang 330006, China

**Keywords:** non-centrosomal MTOCs, Cx26, *GJB2*, pillar cell, camsap2

## Abstract

Mutations in the *GJB2* gene account for approximately 20–50% of all non-syndromic hereditary deafness cases. The malformed organ of Corti (OC) was observed in different Cx26-null mouse models, which was mainly caused by the developmental arrest of pillar cells (PCs). However, the mechanism of developmental abnormalities in PCs caused by Cx26 deletion is still unclear. In this study, the ultrastructure of PCs at different postnatal days was observed in Cx26-null mice. Knockout of cochlear Cx26 led to the malformed assembly of non-centrosomal microtubule-organizing centers (MTOCs) far from the centrosome rather than near the centrosome. Additionally, the microtubule (MT) arrays emitted by abnormal non-centrosomal MTOCs were significantly reduced. In addition, we found that the protein expression of calmodulin-regulated, spectrin-associated protein2 (camsap2), a microtubule minus-end targeting protein associated with the organization of non-centrosomal MTs, was decreased in juvenile PCs in the Cx26-null group. Our results indicated that the malformation of non-centrosomal MTOCs in cochlear PCs might lead to the corresponding MTs’ failure to be captured and anchored in Cx26-null mice, which results in the deformity of OC. Additionally, this abnormal developmental process might be correlated with the reduced expression of camsap2 caused by Cx26 deletion in the early developmental stage.

## 1. Introduction

Mutations in the *GJB2* (encoding Cx26) gene account for about 20–50% of all non-syndromic hereditary deafness cases [1]. Cx26-formed gap junctions widely exist in the supporting cells (SCs) of cochlear epithelium and connective tissues, which play a vital role in the development of the mammalian inner ear [2,3]. Malformation of the organ of Corti (OC) (including the collapse of the tunnel of Corti (TC) and the disappearance of Nuel’s space) is observed in Cx26-null and Cx26-R75W mutation mouse models, which account for severe hearing loss [1,4]. The deformity of OC may be due to cytoskeleton disorder in pillar cells (PCs) because significantly decreased numbers of microtubules (MTs) in PCs are identified in Cx26-null mice [4,5,6]. However, the mechanism of developmental abnormalities in PCs caused by Cx26 deletion is still unclear.

MTs are assembled from heterodimers of polarized subunits, α- and β-tubulin [7]. They form a major cytoskeleton to mediate intracellular transport and maintain the cellular structure by their intrinsic polarity [7,8]. MTs have a relatively stable minus end and a more dynamic plus end. The minus ends can be anchored at the microtubule-organizing centers (MTOCs), where microtubule nucleators are recruited. Additionally, the plus ends can grow and disassemble rapidly. Through this process, cells are able to arrange microtubule arrays in an ordered orientation during the cell cycle and cell differentiation [7,8,9].

**Figure 1:**
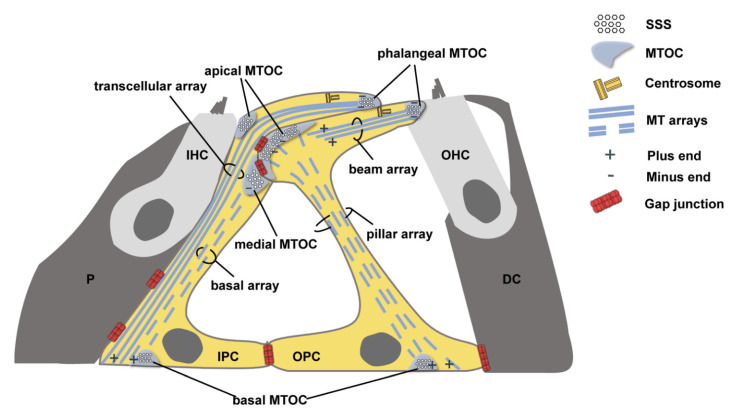
Schematic diagram showing the non-centrosomal MTOCs and the arrangement of MT arrays in PCs. The surfoskelosomes (SSSs) are assembled at MTOCs of PCs and connect MT ends to the cell surface. MTOC: microtubule-organizing center, SSS: surfoskelosome, MT: microtubule, IHC: inner hair cell, OHC: outer hair cell, IPC: inner pillar cell, OPC: outer pillar cell, P: inner phalangeal cell, DC: Deiters’ cell.

In proliferating animal cells, MT nucleation and anchorage occur in the centrosome. However, in most differentiated animal cells, such as epithelial cells, MTs are anchored in non-centrosomal MTOCs [10]. Mature PCs are differentiated epithelial cells. Mature inner pillar cells (IPCs) have four cell-surface-associated MTOCs (Figure 1). One of them is apically located at the tip of the cell’s phalangeal process near the centrioles. The other three are, respectively, situated in apical, medial, and basal positions away from the centrioles. The MT bundles of IPCs consist of two closely aligned MT arrays (transcellular array and basal array) (Figure 1). The transcellular array is anchored at the phalangeal MTOC, while the basal array is anchored at the medial MTOC. Both of them span the longitudinal axis of the IPC downwards to the basal plasma membrane [11]. Mature outer pillar cells (OPCs) have three cell-surface-associated MTOCs, respectively, located at the tip of the cell’s phalangeal process and apical and basal sites. Two MT arrays are, respectively, anchored at the phalangeal MTOC (beam array) and the apical MTOC (pillar array) (Figure 1). In mature OPCs, the beam array spans parallel to the apical cell surface while the pillar array extends parallel to the cell’s apicobasal axis [12]. Under the electron microscope, concentrations of specialized cytoskeletal materials and junctions, called surfoskelosomes (SSSs), are observed at MTOCs of PCs and connect MT ends to the cell surface [11,12].

To further explore the mechanism of cytoskeletal dysplasia in cochlear PCs caused by Cx26 deletion, we used Cx26-null mice to observe MTOCs and the corresponding MTs under the electron microscope. We found impaired degradation of organelles, abnormal assembly of medial and basal SSSs, and decreased numbers of the corresponding MT arrays in cochlear PCs in Cx26-null mice. In addition, we found that the protein expression level of calmodulin-regulated spectrin-associated protein2 (camsap2), which is involved in MT anchoring, was reduced in PCs in Cx26-null groups. Thus, we speculated that the abnormal assembly of non-centrosomal MTOCs may be involved in cytoskeletal dysplasia in PCs caused by Cx26 deletion. Additionally, camsap2 may play a vital role in the developmental process of cochlear PCs.

## 2. Materials and Methods

### 2.1. Mouse Models

Transgenic mouse models Cx26^loxP/loxP^ and Rosa26-CreER were provided by Prof. Xi Lin at Emory University. Tamoxifen-inducible Rosa26-CreER; Cx26^loxP/loxP^ mice were generated by crossbreeding Rosa26-CreER with Cx26^loxP/loxP^ mice. Mouse genotyping was performed by PCR amplification of tail genomic DNA. Details of breeding and genotyping of the mice were given in our previous paper [5]. The genotyping primers were as follows:

Cx26 (F): 5′-ACAGAAATGTGTTGGTGATGG-3′,
Cx26 (R): 5′-CTTTCCAATGCTGGTGGAGTG-3′,
Rosa26Cre(F): 5’-AGCTAAACATGCTTCATCGTCGGTC-3’,
Rosa26Cre(R): 5’-TATCCAGGTTACGGATATAGTTCATG-3’.

To obtain time-specific deletion of Cx26-null mice, a dose of TMX 402 (T5648-1G, Sigma-Aldrich, St Louis, MO, USA) (total dose of 1.5 mg/10 g body weight for two days) was injected subcutaneously at P0 and P1. Rosa26-CreER; Cx26^loxP/loxP^ mice were used as experimental groups, and the littermate Cx26^loxP/loxP^ mice were used as controls.

All mice were raised in the specific-pathogen-free Experimental Animal Centre of Huazhong University of Science and Technology. The experimental process was conducted in accordance with the policies of the Committee on Animal Research of Tongji Medical College, Huazhong University of Science and Technology.

### 2.2. Transmission Electron Microscopy (TEM)

The detailed procedure has been described in our previous studies [5,6]. Three mice were used in the control and Cx26-null groups, respectively. Mice were anesthetized and sacrificed at P10 and P18. The cochleae were fully fixed in a mixture of 2% paraformaldehyde and 2.5% glutaraldehyde in 0.1 M PB, then decalcified in 10% disodium EDTA (pH 7.2) and post-fixed for 1 h in 1% osmium tetroxide. After dehydration, the cochlea was embedded in resin, sectioned (80 nm in thickness), and stained with uranyl acetate and lead citrate. Then, ultrathin sections were observed by using electron microscopy examination (FEI Tecnai G2 20 TWIN; Hillsboro, OR, USA).

### 2.3. RNA Preparation and Real-Time Quantitative Polymerase Chain Reaction (RT-qPCR)

The detailed process has been described in our previous studies [13,14]. RT-qPCR was performed to determine the transcriptional expression level of the following genes: GAPDH, AKAP450, Cdk5rap2, GM130, Camsap1, Camsap2, Camsap3, Clasp1, Clasp2, and Mtcl1. Mice were anesthetized and sacrificed at P6. Then, the cochlear basilar membrane was dissected carefully on ice. For control and experimental groups, there were six biological replicates, respectively. Total RNA was extracted from the membranous labyrinths tissues using an RNAprep Pure Tissue Kit (Tiangen Biotech Co. Ltd., Beijing, China). Then, cDNA was obtained by using a PrimeScript RT Reagent Kit with gDNA eraser (Takara Bio Inc., Shiga, Japan). RT-PCR was performed in a LightCycler 480 instrument (Roche, Basel, Switzerland). Analysis of the relative level of mRNA was performed according to the standard 2^−^^∆∆C^^T^ method. The following primers were used for RT-qPCR:

GAPDH (F): 5’-GAAGGTCGGTGTGAACGGAT-3’;
GAPDH (R): 5’-CTCGCTCCTGGAAGATGGTG-3’;
AKAP450 (F): 5’-AGCCCAATATGATGGGGACAT-3’;
AKAP450 (R): 5’-CTGAGAACTTTCCGAGCAGAG-3’;
Cdk5rap2 (F): 5’-CTCGGGGATGGAAGAGGAAG-3’;
Cdk5rap2 (R): 5’-AAGCCAGAAGTGTCACTGATG-3’;
GM130 (F): 5’-GGGCCTCACATCTTCCAACAT-3’;
GM130 (R): 5’-GACACCAGGATGCCTATGGTC-3’;
Camsap1 (F): 5’-CCTATGGCCTAGACAACATCCC-3’;
Camsap1 (R): 5’-ATAACGGGTGGCTTAATGTGC-3’;
Camsap2 (F): 5’-GGCCAAAATCGCCTGCAATC-3’;
Camsap2 (R): 5’-GTCTGTGTAAAATGGGTCGCC-3’;
Camsap3 (F): 5’-CCACTGCGGAGGACTTTCC-3’;
Camsap3 (R): 5’-ACTGATCGGTGTAGAAGGGTT-3’;
Clasp1 (F): 5’-CCTTGAGCACGACCAGACC-3’;
Clasp1 (R): 5’-CGATTTGCGCCTTGAACCG-3’;
Clasp2 (F): 5’-TGGATGGAAATAGGCCGTCG-3’;
Clasp2 (R): 5’-CCTCCAACCTTAGGGCCAC-3’;
Mtcl1 (F): 5’-AGCCTGAAAGTGGCGGAAAC-3’;
Mtcl1 (R): 5’-CGAGTTCGTGGTGTAATCTGAC-3’.

### 2.4. Immunofluorescence Staining

The detailed procedure has been previously described in our studies [5,6,14]. A total of 3~6 mice were used in the control and Cx26-null groups, respectively. Mice were anesthetized and sacrificed at P6, P10, and P14. Then, the cochleae were fully fixed in 4% paraformaldehyde in 0.01 M PBS at room temperature for 2 h. The apical basement membranes were dissected and then incubated in a blocking solution (10% donkey serum with 0.1% Triton X-100) for 1 h at room temperature. After blocking, the samples were incubated with polyclonal rabbit anti-Camsap2 antibodies (1:200 dilution, 17880-1-AP, Proteintech, Wuhan, China) and monoclonal rabbit anti-α-tubulin antibodies (1:200 dilution; ab179484; Abcam, Cambridge, UK), diluted in 0.01M PBS with 0.3% Triton X-100 overnight at 4 °C. The next day, the samples were placed at room temperature for 1 h and then washed four times with 0.01 M PBST (10 min each time). Samples were then stained by the Alexa Fluor 647-conjugated donkey anti-rabbit IgG (1:200 dilution, ANT032, Antgene Biotechnology Company Ltd., Wuhan, China) for 2 h at room temperature. DAPI (C1005, Beyotime Biotechnology, Shanghai, China) and phalloidin (40736ES75; Yeasen, Shanghai, China) were used for nuclear and F-actin staining, respectively. Images were captured with a laser scanning confocal microscope (Nikon, Tokyo, Japan). The three-dimensional reconstruction images were produced with the NIS-Element software (Nikon, Tokyo, Japan). The immunolabeling of acetylated α-tubulin and camsap2 was quantified from original images, each taken at ×60 magnification in identical conditions of HV, offset, and laser intensity. The images were analyzed with ImageJ software, and the relative fluorescence was quantified by normalizing the ratio of fluorescence of PCs in the Cx26-null group to the average fluorescence of PCs in the corresponding control group.

### 2.5. Data Analysis

GraphPad Prism (Version 8.0.1, GraphPad Software Inc., La Jolla, CA, USA) was used for data analysis. Two-tailed unpaired Student’s *t*-tests were performed according to different comparisons between groups. A *p*-value < 0.05 was considered statistically significant. All data were presented as means ± SEM, and *p* < 0.05 was considered to be statistically significant.

## 3. Results

### 3.1. Impaired Degradation of Organelles in PCs and Hypertrophy of PCs in Cx26-Null Mice

**Figure 2:**
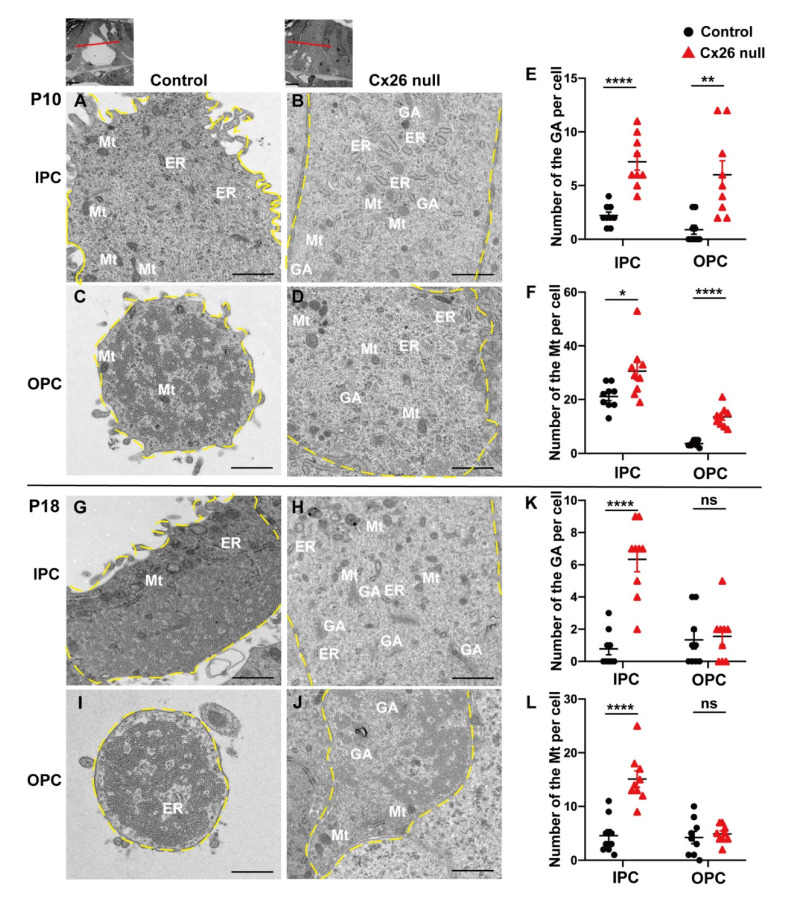
Impaired degradation of organelles in PCs and hypertrophy of PCs in Cx26-null mice. (**A**–**D**) TEM cross-sections obtained from the middle turn in the cochlea show the ultrastructure of IPCs and OPCs at P10; (**E**,**F**) Quantifications of the changes of the Mt and GA at the middle turn in the control and Cx26-null groups (9 IPCs and 9 OPCs from 3 mice in each group) at P10. The number of the GA: 2.2 ± 0.3 (control) vs. 7.2 ± 0.8 (Cx26 null) in IPCs, 0.9 ± 0.4 (control) vs. 6.0 ± 1.3 (Cx26 null) in OPCs. The number of the Mt: 21.1 ± 1.5 (control) vs. 30.6 ± 3.3 (Cx26 null) in IPCs, 3.7 ± 0.4 (control) vs. 13.6 ± 1.2 (Cx26 null) in OPCs. (**G**–**J**) TEM cross-sections obtained from the middle turn in the cochlea show the ultrastructure of IPCs and OPCs at P18. (**K**,**L**) Quantifications of the changes of the Mt and GA at the middle turn in the control and Cx26-null groups (9 IPCs and 9 OPCs from 3 mice in each group) at P18. The number of the GA: 0.8 ± 0.4 (control) vs. 6.3 ± 0.8 (Cx26 null) in IPCs, 1.3 ± 0.6 (control) vs. 1.6 ± 0.5 (Cx26 null) in OPCs. The number of the Mt: 4.6 ± 1.1 (control) vs. 15.1 ± 1.5 (Cx26 null) in IPCs, 4.2 ± 1.1 (control) vs. 4.9 ± 0.5 (Cx26 null) in OPCs. Small images (scale bars: about 10 μm) at the top show the assessed levels of these horizontal sections. Hypertrophic cell body (broken yellow lines) of PCs was observed in Cx26-null group (**D**,**H**,**J**). Data are expressed as mean with SEM; * *p* < 0.05, ** *p* < 0.01, **** *p* < 0.0001, ns: no significant difference, TEM: transmission electron microscopy, P: postnatal, IPC: inner pillar cell, OPC: outer pillar cell, Mt: mitochondria, GA: Golgi apparatus, ER: endoplasmic reticulum. Bars: about 1 μm.

The inner ear of mice gradually matures after birth, and the onset of hearing is around P14. During this period, PCs differentiate into the polarized epithelial cells, and a TC is formed between IPCs and OPCs [1,5]. In this study, we found that a mass of organelles, including mitochondria, Golgi apparatus (GA), and endoplasmic reticulum (ER), remained in PCs’ cytoplasm in the Cx26-null mice at P18 compared with the control group (Figure 2G–J). In IPCs’ cytoplasm at P18, organelles were mainly gathered in the outer side of cells in the control group, whereas lots of mitochondria, GA, and ER existed throughout the cytoplasm in the Cx26-null mice (Figure 2G,H). Statistical analysis showed that the number of Mt and GA in IPCs was increased in the Cx26-null group at P10 (GA: 2.2 ± 0.3 (control) vs. 7.2 ± 0.8 (Cx26 null), *p* < 0.0001; Mt: 21.1 ± 1.5 (control) vs. 30.6 ± 3.3 (Cx26 null), *p* < 0.05) and P18 (GA: 0.8 ± 0.4 (control) vs. 6.3 ± 0.8 (Cx26 null), *p* < 0.0001; Mt: 4.6 ± 1.1 (control) vs. 15.1 ± 1.5 (Cx26 null), *p* < 0.0001) (Figure 2E,F,K,L). The number of Mt and GA in OPCs was also increased in the Cx26-null group at P10 (GA: 0.9 ± 0.4 (control) vs. 6.0 ± 1.3 (Cx26 null), *p* < 0.01; Mt: 3.7 ± 0.4 (control) vs. 13.6 ± 1.2 (Cx26 null), *p* < 0.0001) (Figure 2E,F). There was no statistically significant difference in the number of Mt and GA in the OPCs’ cytoplasm between the two groups at P18 (Figure 2K,L).

In addition, we found morphological changes in PCs in Cx26-null mice at P10 and P18 (Figure 2). The medial cross-section of the cochlea showed rounded OPCs in the control group, while the irregular shape was observed in Cx26-null mice (Figure 2C,D,I,J). The significant hypertrophic cell body of PCs was also observed in the Cx26-null group (Figure 2D,H,J).

### 3.2. MT Arrays Anchored at the MTOCs Away from the Centrosome Rather Than MTOCs near the Centrosome Were Significantly Reduced in the Cx26-Null Group

**Figure 3:**
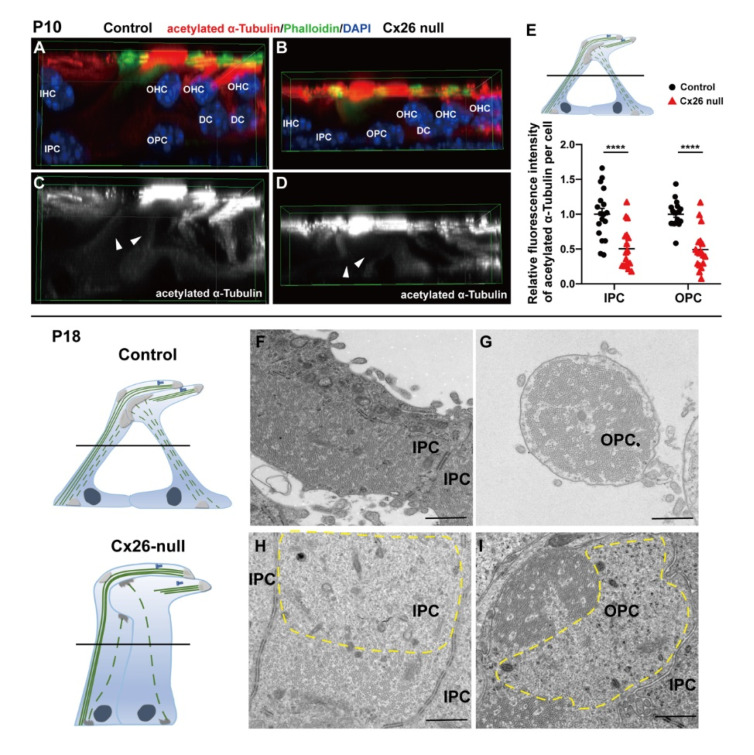
Reduced MT array anchored at non-centrosomal MTOCs away from centrosome in Cx26-null group. (**A**–**D**) Immunofluorescence staining of acetylated α-Tubulin at P10. Three-dimensional reconstruction of apical turn in the cochlea shows the local position of the organ of Corti. (**A**,**C**) width: 70.64 μm, height: 70.64 μm, depth: 35.00 μm. (**B**,**D**) width: 70.64 μm, height: 70.64 μm, depth: 21.00 μm. White arrowheads indicate the MT arrays anchored to non-centrosomal MTOCs away from centrosome. (**E**) Quantification of immunolabeling for acetylated α-Tubulin in IPCs and OPCs from the control and Cx26-null groups (18 IPCs and 18 OPCs from 3 mice in each group) at P10. Schematic diagrams (top) display the assessed levels of the PCs. The relative fluorescence intensity of acetylated α-Tubulin: 1.00 ± 0.09 (control) vs. 0.50 ± 0.07 (Cx26 null) in IPCs, 1.00 ± 0.04 (control) vs. 0.49 ± 0.07 (Cx26 null) in OPCs—data are expressed as mean with SEM, **** *p* < 0.0001; (**F**–**I**) TEM cross-sections obtained from the middle turn in the cochlea show MT arrays in IPCs and OPCs at P18. Schematic diagrams (left) display the assessed levels of these cross-sections (right). Cluttered and absent MTs (broken yellow lines) were observed in Cx26-null group (**H**,**I**). P: postnatal, TEM: transmission electron microscopy, MT: microtubule, IHC: inner hair cell, OHC: outer hair cell, IPC: inner pillar cell, OPC: outer pillar cell, DC: Deiters’ cell. Bars: about 1 μm.

In Cx26-null mice, the immunolabeling of acetylation α-Tubulin of the longitudinal MT arrays was decreased at P10 (1.00 ± 0.09 (control) vs. 0.50 ± 0.07 (Cx26 null) in IPCs and 1.00 ± 0.04 (control) vs. 0.49 ± 0.07 (Cx26 null) in OPCs, *p* < 0.0001) (Figure 3A–E). In addition, cluttered and absent MTs that were anchored at the medial MTOC in IPCs or apical MTOC in OPCs were observed in the Cx26-null group at P18 (Figure 3H,I).

**Figure 4:**
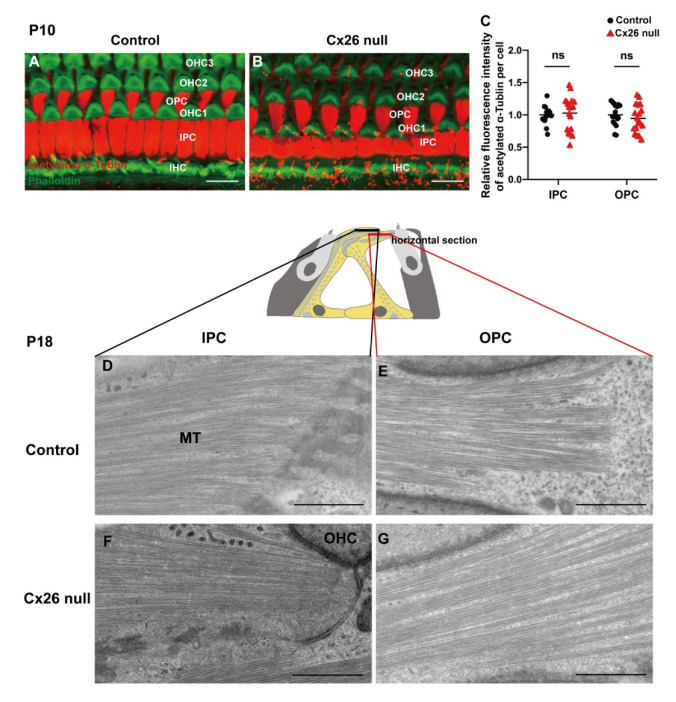
Normal organization of MTs, which are anchored at non-centrosomal MTOCs near centrosome. (**A**,**B**) Immunofluorescence staining of acetylated α-Tubulin in the apical cochlea at P10. (**C**) Quantification of immunolabeling for acetylated α-Tubulin in IPCs and OPCs from the control and Cx26-null groups (18 IPCs and 18 OPCs from 3 mice in each group) at P10. The assessed levels are displayed in plot A,B. The relative fluorescence intensity of acetylated α-Tubulin: 1.00 ± 0.03 (control) vs. 1.03 ± 0.06 (Cx26 null) in IPCs, 1.00 ± 0.04 (control) vs. 0.95 ± 0.05 (Cx26 null) in OPCs—data are expressed as mean with SEM, ns: no significant difference. (**D**–**G**) TEM horizontal sections of PCs obtained from the middle turn in the cochlea at P18. Schematic diagrams (middle) display the assessed levels of (**D**–**G**). P: postnatal, TEM: transmission electron microscopy, MT: microtubule, IHC: inner hair cell, OHC: outer hair cell, IPC: inner pillar cell, OPC: outer pillar cell. Bars: about 10 μm (**A**,**B**), about 1 μm (**D**–**G**).

Statistical analysis showed there was no significant difference in the relative fluorescence intensity of acetylated α-Tubulin in the transcellular array in IPCs and the beam array in OPCs (1.00 ± 0.03 (control) vs. 1.03 ± 0.06 (Cx26 null) in IPCs, 1.00 ± 0.04 (control) VS 0.95 ± 0.05 (Cx26 null) in OPCs) between two groups (Figure 4A–C). TEM horizontal sections of PCs showed a similar MT arrangement of the transcellular array in IPCs and the beam array in OPCs between the two groups (Figure 4D–G).

### 3.3. Ultrastructural Changes of PCs’ Non-centrosomal MTOCs in Cx26-Null Mice

**Figure 5:**
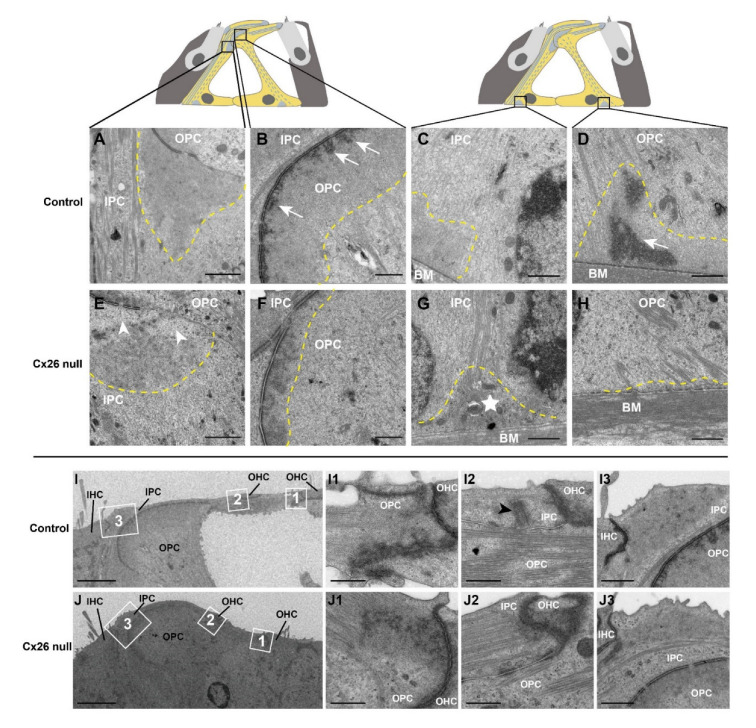
Ultrastructural changes of PCs’ non-centrosomal MTOCs in Cx26-null mice. (**A**–**H**) TEM longitudinal sections of PCs at P18. The assessed positions are displayed in the schematic diagrams (top). The SSSs at non-centrosomal MTOCs far from centrosome are indicated by broken yellow lines. Loose fibrous meshwork of SSSs was observed in Cx26-null group (broken yellow lines, **E**–**H**). Absent fibrous materials of the medial SSS in the IPC are indicated by white arrowheads. Dense tufts of SSSs highlighted by white arrows were missing in Cx26-null group. Phagocytosis of the basal SSS is indicated by a white star. (**I**,**J**) TEM longitudinal sections show the ultrastructure at non-centrosomal MTOCs near centrosome at P18. (I1, I2, J1, J2) Magnification of positions 1 and 2 in (**I**,**J**), respectively, shows the ultrastructure of phalangeal non-centrosomal MTOCs; (I3, J3) Magnification of position 3 in (**I**,**J**), respectively, shows the ultrastructure of apical non-centrosomal MTOCs in IPCs. A centriole is indicated by a black arrowhead. SSS: surfoskelosome, P: postnatal, TEM: transmission electron microscopy, IHC: inner hair cell, OHC: outer hair cell, IPC: inner pillar cell, OPC: outer pillar cell, BM: basilar membrane. Bars: about 1 μm (**A**–**H**), about 5 μm (**I**,**J**), about 500 nm (I1, I2, J1, J2), about 1 μm (I3, J3).

At P18, ultrastructural changes of non-centrosomal MTOCs were detected in PCs in the Cx26-null group. In the control group, OPCs’ apical SSSs and IPCs’ medial SSSs were assembled by compact and dense fibrous meshwork (Figure 5A,B). However, these materials became loose, and some dense tufts were absent in Cx26-null mice (Figure 5E,F). Additionally, the adjacent MTs became cluttered and could not extend in an ordered orientation (Figure 5E,F). In addition, degradation or disappearance of the basal SSSs was observed in PCs without expression of Cx26 (Figure 5G,H).

The assembly of the phalangeal SSSs in PCs and the apical SSSs in IPCs does not seem to be affected. There was no significant difference in the ultrastructural structures between the two groups. Additionally, the adjacent MTs were orderly anchored at MTOCs (Figure 5I1–I3,J1–J3).

### 3.4. No Changes Were Found in the mRNA Expression Level of Non-Centrosomal MT Nucleation and Anchorage-Associated Proteins

**Figure 6:**
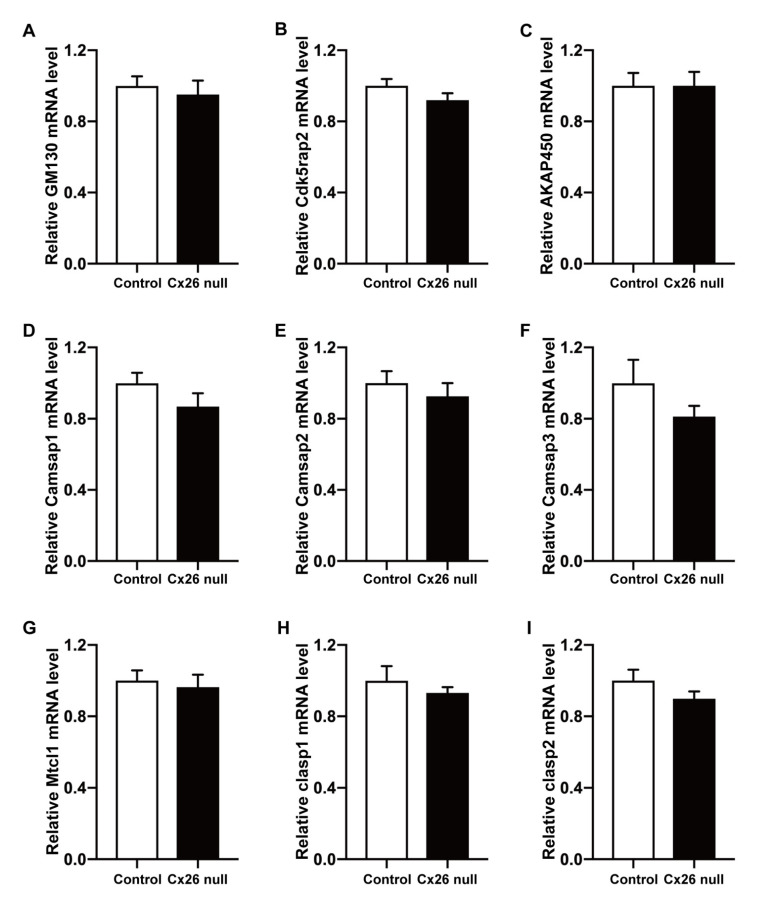
No changes were found in the mRNA expression levels of non-centrosomal MT nucleation- and anchorage-associated proteins. (**A**–**I**) The mRNA expression levels of GM130, Cdk5rap2, AKAP450, Camsap1, Camsap2, Camsap3, Mtcl1, Clasp1, and Clasp2 in the cochlear basilar membrane at P6. There was no significant difference between the control and Cx26-null groups (6 mice in each group, *p* > 0.05).

Many kinds of proteins have been reported to be involved in the nucleation and anchorage of non-centrosomal MTOCs and the assembly and elongation of MTs. Thus, we used RT-qPCR to detect the mRNA expression level of the associated proteins, including nucleators (AKAP450, Cdk5rap2, GM130) and MT-anchoring factors (Camsap1, Camsap2, Camsap3, Clasp1, Clasp2, and Mtcl1) in the cochlear basilar membrane at P6. We did not find a statistically significant difference in mRNA level of the above-detected proteins between the two groups (6 mice in each group, *p* > 0.05) (Figure 6).

### 3.5. Reduced Protein Expression Level of Camsap2 in PCs in the Cx26-Null Group

**Figure 7:**
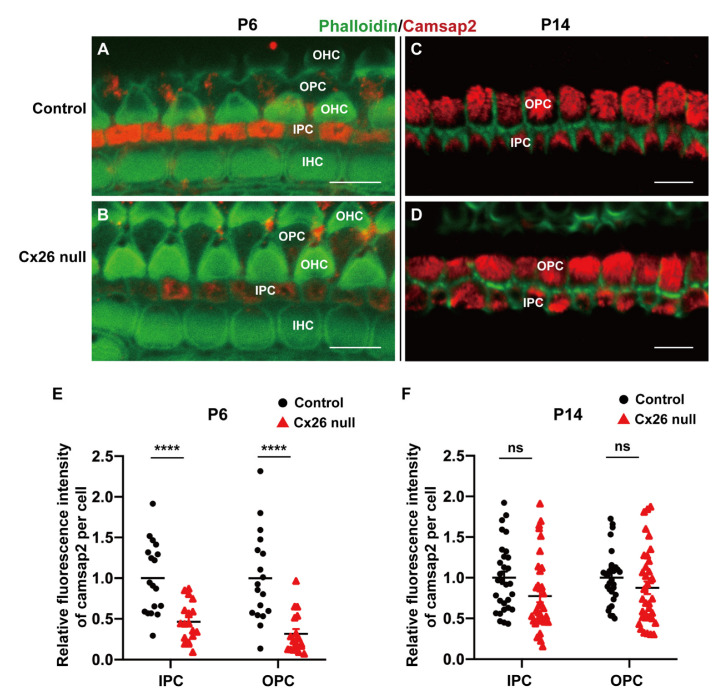
Reduced protein expression level of camsap2 in PCs in Cx26-null group. (**A**,**B**) The immunofluorescence staining of camsap2 in the apical cochlea at P6. (**C**,**D**) The immunofluorescence staining of camsap2 in the apical cochlea at P14. (**E**) Quantification of immunolabeling for camsap2 in IPCs and OPCs from the control and Cx26-null groups (18 IPCs and 18 OPCs from 3 mice in each group) at P6. The assessed levels are displayed in plot (**A**,**B**). The relative fluorescence intensity of camsap2: 1.00 ± 0.10 (control) vs. 0.46 ± 0.06 (Cx26 null) in IPCs, 1.00 ± 0.13 (control) vs. 0.32 ± 0.06 (Cx26 null) in OPCs—data are expressed as mean with SEM, **** *p* < 0.0001. (**F**) Quantification of immunolabeling for camsap2 in IPCs and OPCs from the control and Cx26-null groups (30~36 IPCs and 30~36 OPCs from 5~6 mice in each group) at P14. The assessed levels are displayed in plot (**C**,**D**). The relative fluorescence intensity of camsap2: 1.00 ± 0.08 (control) vs. 0.77 ± 0.08 (Cx26 null) in IPCs, 1.00 ± 0.06 (control) vs. 0.88 ± 0.08 (Cx26 null) in OPCs—data are expressed as mean with SEM, ns: no significant difference; P: postnatal, IHC: inner hair cell, OHC: outer hair cell, IPC: inner pillar cell, OPC: outer pillar cell. Bars: about 10 μm (**A**–**D**).

At P6, we found camsap2 was only expressed in the apical sites of PCs between two groups (Figure 7A,B). Quantification of immunolabeling showed the expression levels of camsap2 were reduced in IPCs (1.00 ± 0.10 (control) vs. 0.46 ± 0.06 (Cx26 null), *p* < 0.0001) and OPCs (1.00 ± 0.13 (control) vs. 0.32 ± 0.06 (Cx26 null), *p* < 0.0001) in the Cx26-null group at P6 (Figure 7A,B,E). As the cochlea gradually matured, we found that the protein expression of camsap2 returned to normal (Figure 7C,D and Supplementary Materials Figure S1). There was no statistically significant difference in the fluorescence intensity of camsap2 in PCs’ MTOCs, including medial MTOCs in IPCs, apical MTOCs in OPCs (Figure 7F), and both phalangeal MTOCs in PCs at P14 (Supplementary Materials Figure S1).

## 4. Discussion

Cx26 deletion affects the deployment or degradation of cochlear PCs’ organelles, resulting in immature PCs. In the developmental process of the inner ear after birth, the organelles gradually disappear or are rearranged in the PCs‘ cytoplasm. Instead, the assembled MTs exist in almost the entire cellular space to form the cytoskeleton. However, this process was impaired in cochlear PCs in Cx26-null mice. At P18, organelle retention and a hypertrophic cell body were observed in PCs, which indicated that PCs were still in the immature state, like at P10.

Cx26 deletion affects the assembly of non-centrosomal MTOCs far from the centrosome and leads to a reduction in the corresponding MT arrays. In the previous studies, significantly reduced MTs were identified in cochlear PCs in Cx26-null and mutation mice [4,5,6]. However, the mechanism for this has not been illuminated. In our study, we found ultrastructural changes in non-centrosomal MTOCs in both IPCs and OPCs in the Cx26-null group. A loose fibrous meshwork and disappeared dense tufts may indicate the impaired assembly of MTOCs. Especially, the adjacent MTs that should be anchored at these positions became cluttered, and the corresponding MT arrays cannot extend in an ordered orientation, which also indicates the possible dysfunction of non-centrosomal MTOCs in PCs. Interestingly, these changes were only observed at non-centrosomal MTOCs and corresponding MTs far from the centrosome instead of the near centrosome. Numerous studies have suggested that the formation of non-centrosomal MTOCs plays a vital role in the determination of the spatial distribution of MT arrays, which in turn affects cell shape and function [9,15]. Thus, we speculated that the reduced MTs might be caused by the dysfunction of non-centrosomal MTOCs and that the disturbance of the basal and pillar MT arrays in PCs is the main cause of malformation of the OC in Cx26-null mice.

**Figure 8:**
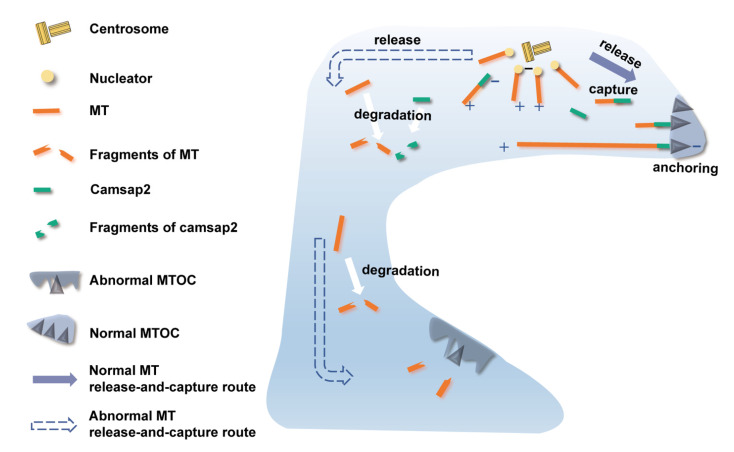
Schematic diagram showing the normal and abnormal MT release-and-capture routes in PCs (taking IPC as an example). MT nucleation occurs at centrosome. Minus ends of MTs are released, successfully captured, and anchored at the non-centrosomal MTOC near the centrosome but fail to be captured and anchored at the non-centrosomal MTOC far from the centrosome. MTOC: microtubule-organizing center, MT: microtubule, IPC: inner pillar cell.

Reduced protein expression of Camsap2 in the early developmental stage may result in cytoskeletal dysplasia in PCs caused by Cx26 deletion. Some studies on cochlear epithelial cells suggested that a microtubule release-and-capture mechanism may be responsible for the arrangement of the apical cell-surface-associated non-centrosomal MT arrays in these cells [16,17]. MT nucleation occurs at the centrosome, but minus ends of MTs are released, captured, and anchored at non-centrosomal MTOCs, which is accompanied by the relocation of the minus-end anchoring-associated proteins [15,16,17] (Figure 8). In this study, we detected the mRNA level of MT nucleators and anchoring factors but found no statistically significant difference between the control and Cx26-null groups. However, we found the reduced protein expression of camsap2 in both IPCs and OPCs, respectively, at P6. At this period, cochlear TC is ready to open in the normal developmental process, and camsap2 is expressed in the apical sites in PCs. Camsap2, a member of the calmodulin-regulated, spectrin-associated protein (CAMSAP) family, has been identified as a key factor of non-centrosomal MTOCs to stabilize MT minus ends and mediate MT outgrowth [9,18] (Figure 8). In the process of assembly of non-centrosomal MTs, camsap2 is recruited to the centrosome from where MTs are released [19]. Additionally, studies on the role of camsap2 showed that depletion of camsap2 resulted in the absence of non-centrosomal MT arrays as well as impaired organelle assembly [19,20]. Thus, we speculated that in the early developmental stage of cochlear PCs, MTs released from the centrosome cannot be captured and anchored at non-centrosomal MTOCs far from the centrosome, as is the case for the reduced expression of camsap2 in Cx26-null mice (Figure 8). In addition, the normal formation of non-centrosomal MTOCs far from the centrosome may be correlated with enough camsap2 expression in the period of TC opening because disturbance of non-centrosomal MTOCs existed even though the protein level of camsap2 was normal later. There must be compensatory pathways for the assembly of non-centrosomal MTOCs near the centrosome and the organization of the corresponding MT arrays. Additionally, further research is needed to confirm our speculation.

In conclusion, our results indicated that a loss of cochlear Cx26 could lead to the malformation of non-centrosomal MTOCs and a reduction in the corresponding MTs in cochlear PCs, which may result in the deformation of OC. Additionally, this abnormal developmental process might be correlated with the reduced expression of camsap2 in the early developmental stage, which might be a potential mechanism for severe deafness in Cx26-null mice.

## Data Availability

The data that support the conclusions of our study are included within the article.

## Supplementary Materials

The following supporting information can be downloaded at: https://www.mdpi.com/article/10.3390/biomedicines10061364/s1, Figure S1: No changes found in protein expression level of camsap2 at the non-centrosomal MTOCs near centrosome at P14.
