# Supplementary material for: The Reduction in Microtubule Arrays Caused by the Dysplasia of the Non-Centrosomal Microtubule-Organizing Center Leads to a Malformed Organ of Corti in the Cx26-Null Mouse
Source: Biomedicines. 2022 Jun 9;10(6):1364. doi: 10.3390/biomedicines10061364 (PMC9219875; doi:10.3390/biomedicines10061364)
Supplement: Supplementary file 1 [file biomedicines-10-01364-s001.zip › biomedicines-1734259-supplementary.pdf]

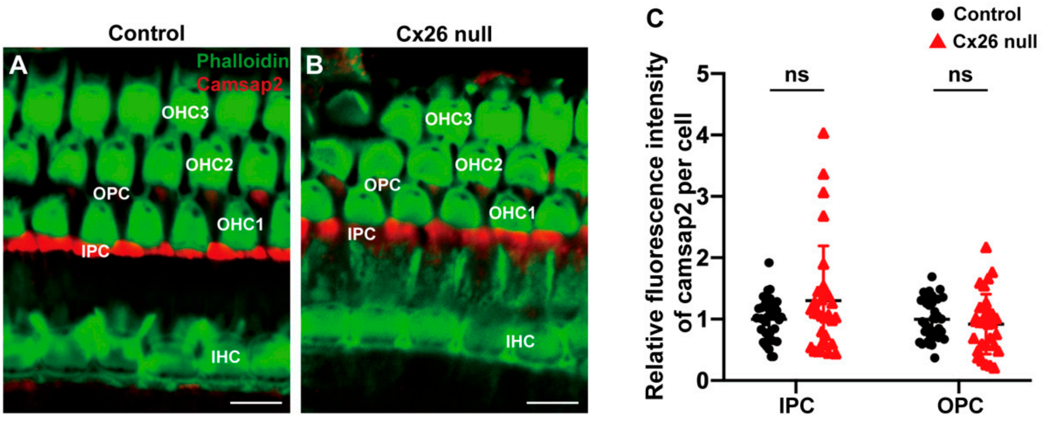

Supplementary Figure S1. No changes found in protein expression level of camsap2 at the non-centrosomal MTOCs near centrosome at P14. (A,B) The immunofluorescence staining of camsap2 in the apical cochlea at P14; (C) Quantification of immunolabeling for camsap2 in IPCs and OPCs from the control and Cx26 null groups (30~36 IPCs and 30~36 OPCs from 5~6 mice in each group) at P14. The assessed levels were displayed in plot A, B. The relative fluorescence intensity of camsap2: 1.00 ± 0.06 (control) VS 1.30 ± 0.16 (Cx26 null) in IPCs, 1.00 ± 0.06 (control) VS 0.92 ± 0.09 (Cx26 null) in OPCs, data were expressed as mean with SEM, ns: no significant difference; P: postnatal, IHC: inner hair cell, OHC: outer hair cell, IPC: inner pillar cell, OPC: outer pillar cell. Bars: about 10 um (A, B).
